# Noise matters: assessing non-auditory health impacts of occupational noise exposure among factory workers

**DOI:** 10.3389/fpubh.2026.1753715

**Published:** 2026-02-18

**Authors:** S. Krishnan, K. Priyasha, Manasi Bhattacharjee, Abhishek Sinha, Benzamin Hanse, Saklain Mustak Alam, Jyotirmoy Kalita

**Affiliations:** Department of Physiology, All India Institute of Medical Sciences, Guwahati, Guwahati, India

**Keywords:** cardiovascular health, cognitive impairment, metabolic function, occupational noise exposure, workers

## Abstract

**Background:**

Industrial noise exposure is a global occupational hazard, with increasing recognition of its deleterious non-auditory effects. Although the auditory consequences of excessive noise are well-known, an emerging body of evidence suggests significant impacts on cognition, cardiovascular and metabolic functions. India with a burgeoning industrial workforce and limited occupational health surveillance, remains under-researched in this domain.

**Objectives:**

This study investigates the physiological and sensory processing impact of occupational noise exposure among industrial workers in Guwahati, a rapidly industrializing region in Northeast India.

**Methods:**

A longitudinal study was conducted involving 590 workers from various factories in Guwahati. Ambient noise levels were recorded using calibrated sound level meters. Workers were assessed before and after their work shifts for sensory processing (via reaction time tests), cardiovascular health (blood pressure and heart rate), metabolic health (blood glucose levels), and stress markers (blood cortisol). Ethical clearance was obtained from the Institutional Ethics Committee.

**Results:**

Noise levels across the industrial settings ranged from 62.2 dB(A) to 91.8 dB(A). High noise levels were particularly noted in batch manufacturing rooms and weighing machine areas. Significant post-shift increases were observed in systolic and diastolic blood pressure (mean increases of 13.2 mmHg and 9.3 mmHg respectively, *p* < 0.001), heart rate (mean increase of 4.5 bpm, *p* < 0.001), visual reaction time (mean increase of 50 ms, *p* < 0.001), and auditory reaction time (mean increase of 50 ms, *p* < 0.001). Cortisol levels also rose significantly (mean increase of 15.8 ng/mL, *p* < 0.001), and elevated blood glucose was observed in a substantial proportion of workers post-shift. Regression analysis demonstrated moderate positive associations between noise exposure and reaction times (visual reaction time: *R*^2^ = 0.48; auditory reaction time: *R*^2^ = 0.32), indicating delayed cognitive responses with increasing noise levels.

**Conclusion:**

The study demonstrates significant non-auditory impacts of occupational noise exposure, suggesting impaired cognition, heightened physiological stress, and acute alterations in metabolic markers among industrial workers. These findings highlight the need for preventive noise management strategies and short-term occupational health monitoring, engineering controls, and routine health surveillance to mitigate these effects.

**Practical applications:**

Our findings demonstrate the need for factories to adopt effective noise control measures such as engineering modifications, administrative controls, and mandatory hearing protection. These steps can reduce workers’ noise exposure, minimizing physiological stress and cognitive impairments, and thus improve overall occupational health and productivity.

## 1 Introduction

Noise pollution has emerged as one of the leading environmental health threats globally, second only to air pollution from particulate matter in terms of burden of disease attributable to environmental risk factors (1). The increasing urbanization and industrialization have intensified noise levels in both living and working environments. In occupational settings, especially in low- and middle-income countries (LMICs), noise pollution remains a pervasive and largely under-regulated hazard. An estimated 250 million workers globally are exposed to hazardous noise levels, defined by the World Health Organization (WHO) and the Occupational Safety and Health Administration (OSHA) as exceeding 85 decibels (dB) over an 8-h time-weighted average (2). In India, studies have suggested that approximately 25% of the industrial workforce experiences exposure levels exceeding 65 dB(A), with many settings surpassing even higher thresholds (3, 4). Traditionally, occupational noise exposure has been associated with auditory effects, particularly noise-induced hearing loss (NIHL). However, a growing body of evidence now indicates that chronic exposure to high-decibel environments also results in substantial non-auditory effects. These include cardiovascular dysfunction, sleep disturbances, metabolic disorders, increased cortisol levels, impaired immune responses, and cognitive dysfunctions (5, 6). Mechanistically, noise exposure leads to activation of the hypothalamic–pituitary–adrenal (HPA) axis and the sympathetic-adrenal-medullary (SAM) system, resulting in increased secretion of stress hormones such as cortisol and adrenaline. This sustained activation can elevate blood pressure, increase heart rate, and impair cognitive performance, particularly in tasks requiring sustained attention and quick decision-making (2, 7). Despite these recognized impacts, there remains a paucity of primary research on the non-auditory effects of occupational noise in the Indian context. This gap is especially pronounced in Northeast India, a region undergoing rapid industrial growth occupational health surveillance and protective regulations. Furthermore, most existing studies are either cross-sectional or limited in sample size, failing to capture dynamic physiological changes due to acute noise exposure. In this context, the present longitudinal study aims to comprehensively evaluate the non-auditory health impacts of workplace noise exposure among industrial workers in Guwahati, Assam. Specifically, the study investigates alterations in cognitive performance using reaction time assessments, cardiovascular parameters including blood pressure and heart rate, and metabolic indicators such as blood glucose and blood cortisol levels. The study seeks to contribute to evidence-based policymaking and reinforce the need for noise mitigation strategies in Indian industrial health frameworks.

## 2 Materials and methods

The present study employed a longitudinal observational design conducted from July 2023 to October, 2023 to evaluate acute physiological and cognitive changes associated with occupational noise exposure among industrial workers. Data collection was conducted over a four-month period, pre- and post-shift measurements were measured to assess immediate effects during a typical workday. The study was carried out in selected industrial units located in and around Guwahati, Assam. Although the broader research project includes multiple industrial sectors, the present analysis focuses specifically on chemical processing manufacturing units with similar production processes, selected to ensure comparability of noise exposure conditions and to characterize typical noise environments within this industrial category. A total of 590 adult workers aged 18–60 years participated, meeting inclusion criteria of at least 6 months of continuous employment and voluntary consent, while individuals with neurological, psychiatric, or cardiovascular disorders, or those on medications affecting cardiovascular or metabolic function, were excluded. Recruitment was facilitated through factory management, and participation was entirely voluntary. The study protocol received approval from the Institutional Ethics Committee (IEC) of AIIMS Guwahati, and all participants provided written informed consent after receiving detailed information about the objectives, procedures, and potential risks in the study.

Data collection was performed on-site within factory premises, with each participant assessed for all the mentioned parameters at the beginning and end of their 8-h shift. Ambient noise levels were measured using calibrated digital sound level meters (SLMs) mounted at shoulder height, positioned away from reflective surfaces to prevent distortion, and averaged over 15-min intervals across operational zones such as E-cutting machine areas, SRP units, cooling sections, batch rooms, and weighing areas. Ambient noise levels were measured using a calibrated digital sound level meter (Model: SIGMA SL101+; Manufacturing country: China; Serial No.: 25107797). Measurements were recorded in A-weighted decibels [dB(A)]. The instrument was operated in hand-held mode and positioned at approximately shoulder height of the workers, away from reflective surfaces, following standard occupational noise measurement guidelines. Physiological parameters, including blood pressure and heart rate, were measured at rest using validated automated sphygmomanometers after a minimum 5-min seated rest, with two readings averaged per time point. Metabolic assessments included capillary blood glucose, measured using calibrated Accu-Chek® Active glucometers, and serum cortisol, quantified via enzyme-linked immunosorbent assay (ELISA) from samples stored at −20 °C, maintaining inter and intra-assay coefficients of variation below 10%. Cognitive performance was evaluated using reaction time tests both visual and auditory administered through standardized computerized tools developed and validated in prior Indian population studies; the mean of five trials was used for analysis.

Statistical analyses were performed using SPSS version 26. Descriptive statistics were computed for all variables, and paired t-tests were applied to compare pre- and post-shift values of blood pressure, heart rate, blood glucose, cortisol, and reaction times. Statistical significance was defined as *p* < 0.05. To explore the relationship between noise exposure and cognitive performance, simple linear regression analyses were conducted with noise level as the independent variable and reaction time as the dependent variable, and the coefficient of determination (*R*^2^) was reported to quantify the strength of associations.

## 3 Results

### 3.1 Noise levels

**Figure 1:**
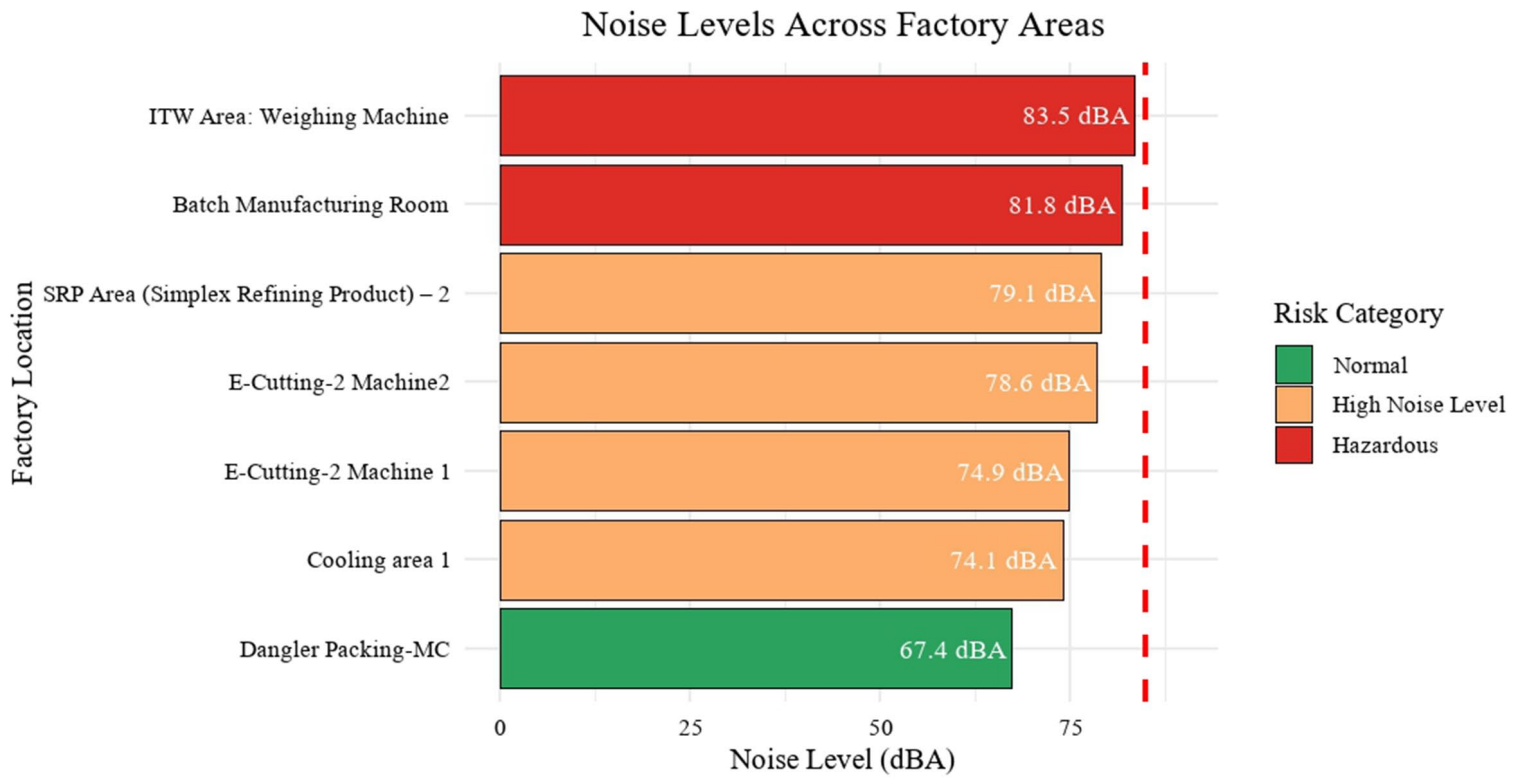
Noise levels across various factory areas.

Noise measurements across the factories demonstrated significant spatial variability, with recorded levels at the seven locations within the company ranging from 67.4 dB(A) to 83.5 dB(A) (Figure 1). The Dangler Packing-MC exhibited the lowest noise exposure at 67.4 dB(A), while the ITW area: weighing machine reached the highest at 83.5 dB(A). Most assessed areas, including both E-Cutting-2 machines, the SRP Area, and the Cooling Area, showed elevated noise measurements exceeding 74 dB(A). Notably, both the ITW Area: Weighing Machine 83.5 dB(A) and the Batch Manufacturing Room [81.8 dB(A)] approached the widely recognized occupational noise exposure threshold of 85 dB(A) as recommended by NIOSH. Apart from Dangler Packing-MC, all locations recorded noise levels above 70 dB(A), indicating that most zones pose a considerable risk for excessive noise exposure. These findings highlight the urgent need for targeted noise reduction measures within the facility to ensure employee safety and compliance with occupational.

### 3.2 Physiological impact of occupational noise exposure

We found that regular exposure to loud noise in the workplace led to clear physiological and cognitive stress responses among workers (Table 1). After completing shifts in high-noise settings, workers demonstrated notable increases in cardiovascular markers: average systolic blood pressure rose from 121.1 ± 4.6 mmHg pre-shift to 134.3 ± 5.7 mmHg post-shift, while diastolic pressure climbed from 79.4 ± 2.8 mmHg to 88.7 ± 3.9 mmHg, representing increases of 10.9% and 11.7%, respectively. Heart rate also increased by 5.5%, from 81.9 ± 27.2 bpm to 86.5 ± 14.6 bpm, indicating acute cardiovascular strain, likely due to persistent sympathetic nervous system activation.

**Table 1:** Comparison of cardiometabolic parameters, reaction time before (Pre) and after (Post) noise exposure.

Variables	Pre (mean ± SD)	Post (mean ± SD)	*p*-value
Systolic blood pressure (mmHg)	121.1 ± 4.6	134.3 ± 5.7	0.001*
Diastolic blood pressure (mmHg)	79.4 ± 2.8	88.7 ± 3.9	0.001*
Heart rate (bpm)	81.94 ± 27.15	86.45 ± 14.55	0.001*
Cortisol	82.7 ± 7.3	98.5 ± 8.7	0.001*
Visual reaction time (ms)	300 ± 150	350 ± 140	0.001*
Auditory reaction time (ms)	310 ± 170	360 ± 140	0.001*

Data expressed in (mean ± SD), paired *t* test was used, *p < 0.05 statistically significant.

**Figure 2:**
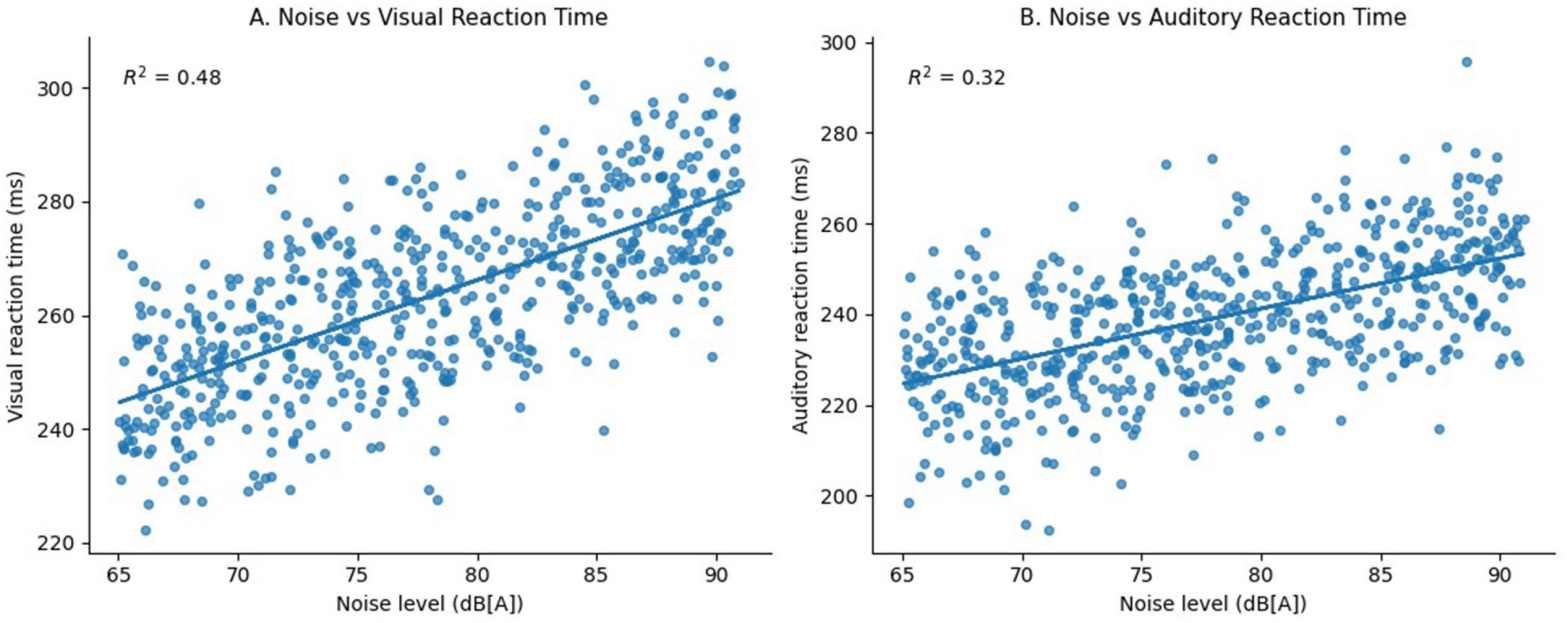
**(A,B)** Relationship between workplace noise level [dB(A)] and auditory reaction time (ms) and visual reaction time (ms) among industrial workers (*n* = 590).

Reaction time changes were equally evident. Workers’ visual reaction times increased from 0.300 ± 0.150 to 0.350 ± 0.140 milli seconds (ms) post-shift, and auditory reaction times from 0.310 ± 0.170 to 0.360 ± 0.140 milli seconds (ms) an approximate 16% slow-down in processing speed and attention (Table 1). The association between environmental noise and reaction time was further examined using regression analysis, which showed moderate positive associations in Figure 2A (noise level vs. visual reaction time; *R*^2^ = 0.48) and Figure 2B (noise level vs. auditory reaction time; *R*^2^ = 0.32), indicating that higher ambient noise levels are associated with delayed cognitive responses.

Noise-induced stress was also reflected in endocrine and metabolic markers. Blood cortisol, a key biomarker of the stress response, rose by 19.1% post-shift (from 82.7 ± 7.3 ng/mL to 98.5 ± 8.7 ng/mL), and 22% of workers exhibited elevated blood glucose (>140 mg/dL) after their shifts (Table 1). Taken together, these findings demonstrate that routine occupational noise exposure places a substantial physiological and cognitive burden on workers. The data visualizations in Figures 2A,B further underscore the close link between noise intensity and cognitive impairment, highlighting the urgent need for workplace noise control, ongoing health monitoring, and targeted interventions to protect worker well-being.

### 3.3 Proportion-based analysis of health outcomes

**Figure 3:**
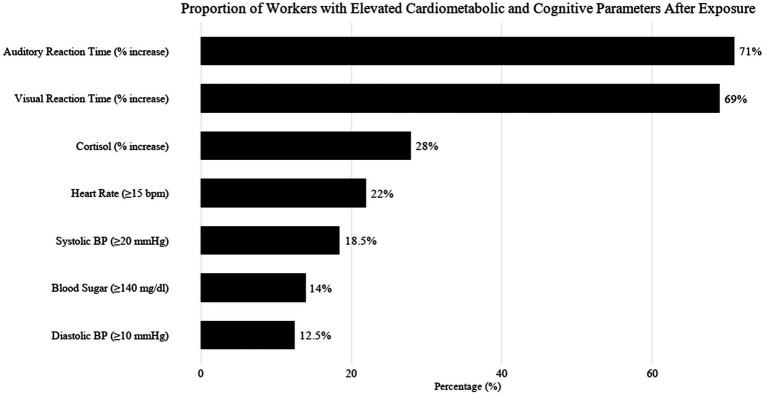
Figure represents proportion of workers with elevated cardiometabolic and cognition parameters postexposure.

Post-exposure assessment of workers revealed significant acute physiological responses linked to occupational noise stress, with a considerable proportion of individuals exhibiting elevated cardiovascular parameters (Figure 3). Specifically, 18.5% of workers experienced an increase in systolic blood pressure (SBP) by ≥20 mmHg, and 12.5% showed a diastolic blood pressure (DBP) rise of ≥10 mmHg following their work shift. Additionally, 22% of participants had an elevated heart rate exceeding 15 beats per minute (bpm) compared to their baseline values. These cardiovascular responses indicate a state of acute autonomic activation, likely mediated by increased sympathetic tone due to prolonged noise exposure. Such changes are consistent with established stress pathways, including heightened vasoconstriction, increased cardiac output, and systemic vascular resistance all reflect acute cardiovascular stress responses observed during noise exposure.

In addition to cardiovascular strain, marked neurocognitive and metabolic alterations were observed. A substantial proportion of workers exhibited delayed sensory and motor responses, with 71% showing increased auditory reaction time and 69% displaying elevated visual reaction time. These changes suggest compromised central processing speed and cognitive efficiency, likely resulting from mental fatigue, attention deficits, or transient dysfunction in auditory and visual pathways. The auditory and visual reaction time delays are particularly concerning in industrial environments where swift decision-making and rapid responses are critical for operational safety. From an endocrine and metabolic standpoint, 28% of workers demonstrated increased cortisol levels post-exposure, reflecting overactivation of the hypothalamic–pituitary–adrenal (HPA) axis. Elevated cortisol is a well-documented biomarker of psychological and physiological stress and is known to influence blood pressure, immune function, and glucose metabolism. Correspondingly, 14% of the participants recorded blood sugar levels exceeding 140 mg/dL, suggestive of stress-induced hyperglycaemia, potentially due to cortisol-mediated hepatic gluconeogenesis and temporary insulin insensitivity.

These findings collectively underscore the multi-system burden of occupational noise exposure, which extends beyond auditory damage to include cardiovascular strain, cognitive impairment, and metabolic disruption. In high-decibel industrial settings where noise levels may surpass occupational exposure limits, the observed physiological disturbances emphasize the urgent need for comprehensive noise hazard management. This includes regular health surveillance, engineering controls, implementation of noise-reducing technologies, and worker education on stress mitigation strategies as part of an integrated approach to industrial health and safety.

## 4 Discussion

The findings from our study reaffirm that occupational noise exposure functions as a significant multi-system health hazard. While the auditory consequences of noise exposure have been widely acknowledged, our data provide compelling evidence for its acute non-auditory physiological and cognitive impacts in industrial workers. Specifically, significant post-shift elevations were observed in blood pressure, heart rate, blood cortisol, blood glucose, and reaction time performance (2, 8). The increase in systolic and diastolic blood pressure, coupled with a rise in heart rate, suggests activation of the sympathetic-adrenal-medullary (SAM) axis, likely due to noise-induced stress. Similarly, Babisch (8) and van Kempen et al. (9) documented that prolonged exposure to noise, particularly above 70 dB(A), is associated with hypertension and ischemic heart disease, especially in occupational settings. Cognitive performance, assessed using visual and auditory reaction times, showed significant associations with workplace noise exposure. Regression analyses demonstrated moderate positive associations between ambient noise levels and delayed reaction times (*R*^2^ = 0.48 for visual and *R*^2^ = 0.32 for auditory), indicating that higher noise levels are associated with slower cognitive responses. These findings are consistent with prior evidence linking noise exposure to impaired cognitive performance (Clark and Paunovic (10)). Blood cortisol, a well-validated biomarker of physiological stress, showed substantial post-exposure elevation in our study. This suggests heightened hypothalamic–pituitary–adrenal (HPA) axis activity, a hallmark of acute stress responses. Our findings corroborate those by Babisch (11) and Seidler et al. (12), who identified significant associations between noise exposure and elevated cortisol levels in both occupational and residential settings. A particularly novel and concerning finding was the acute rise in blood glucose levels in 22% of participants post-shift, with values exceeding 140 mg/dL. While temporary hyperglycemia may be part of an acute stress response, the observed elevations likely represent short-term stress-related metabolic responses or pre-diabetic states. Prior research has suggested that stress-mediated increases in cortisol and catecholamines may impair insulin sensitivity, leading to transient or even chronic metabolic dysregulation (13–15). There is emerging evidence that prolonged exposure to noise can increase the risk for type 2 diabetes, possibly through chronic stress pathways and circadian rhythm disruption. These findings are consistent with global evidence on the non-auditory health effects of noise exposure Basner et al. (2) and the Environmental Noise Guidelines World Health Organization (1). Our study also fills a critical evidence gap by contextualizing these effects within the Indian industrial workforce, particularly in the under-researched region of Northeast India, where baseline health data and occupational safety protocols are often lacking. The proportion-based analysis further highlighted the public health relevance of our findings. Notably, 18.5% of workers experienced a systolic blood pressure increase >20 mmHg, 12.5% had diastolic increases >10 mmHg, 28% showed heart rate elevations >15 bpm, and an alarming 71% had cortisol levels above the upper reference range. These findings are not only statistically significant but also clinically relevant, warranting immediate occupational health interventions (9, 11). Given the same-day pre-post study design, the observed changes represent acute physiological responses to occupational noise exposure and should not be interpreted as evidence of chronic or long-term cardiovascular or metabolic disease risk. While the present findings reflect acute physiological responses to occupational noise exposure, repeated exposure to similar stress responses may potentially influence homeostatic regulation over time, a hypothesis that warrants confirmation through longitudinal studies. In light of this, there is a pressing need to integrate noise monitoring into workplace health and safety frameworks (19, 20). Implementing engineering controls, enforcing regulatory compliance, and promoting worker awareness are key steps toward mitigating the non-auditory effects of noise exposure.

## 5 Conclusion

Occupational noise exposure in industrial settings has far-reaching health implications beyond hearing loss. This study establishes a clear association between elevated noise levels and adverse changes in blood pressure, heart rate, cognitive performance, and metabolic stress indicators. Recognizing noise as a systemic hazard mandates comprehensive workplace safety reforms.
This study demonstrates that occupational noise exposure in industrial settings acutely disrupts key aspects of human physiology, with far-reaching consequences for worker health and safety. Specifically, we found that elevated workplace noise leads to significant rises in blood pressure and heart rate, indicating pronounced cardiovascular stress responses. Higher blood cortisol levels in noise-exposed workers further point to the activation of the hypothalamic–pituitary–adrenal (HPA) axis and an acute neuroendocrine stress reaction, while increased blood glucose concentrations signal immediate metabolic disturbances (2, 13, 16). These rapid physiological perturbations, if repeatedly experienced, may have implications for worker health that require confirmation through longitudinal studies. Beyond these physiological impacts, our findings reveal that occupational noise exposure impairs cognitive performance, as evidenced by slower reaction times and reduced attention, corroborating international reports on the detrimental neurocognitive effects of noise (10, 17). Mechanistic insights from recent multidisciplinary research suggest that these effects are mediated via the activation of the HPA axis and the sympathetic nervous system, resulting in stress responses that disturb homeostatic balance and cognitive processing (13, 18). Taken together, this form of evidence highlights the urgent need for noise mitigation strategies and comprehensive regulatory frameworks to protect both the physiological and mental health of industrial workers, especially in regions facing rapid industrialization and increased occupational noise (1, 2). A multidisciplinary, evidence-based approach to workplace noise management is essential to secure the long-term well-being of the industrial workforce (21).

## Data Availability

The raw data supporting the conclusions of this article will be made available by the authors, without undue reservation.
